# Role of FOXO3a Transcription Factor in the Regulation of Liver Oxidative Injury

**DOI:** 10.3390/antiox11122478

**Published:** 2022-12-16

**Authors:** Hong Jin, Li Zhang, Jun He, Min Wu, Li Jia, Jiabin Guo

**Affiliations:** 1 Center for Disease Control and Prevention of PLA, Beijing 100071, China; 2 School of Public Health, China Medical University, Shenyang 110122, China

**Keywords:** oxidative stress, FOXO3a transcription factor, liver injury, mitochondria, cell survival, cell death

## Abstract

Oxidative stress has been identified as a key mechanism in liver damage caused by various chemicals. The transcription factor FOXO3a has emerged as a critical regulator of redox imbalance. Multiple post-translational changes and epigenetic processes closely regulate the activity of FOXO3a, resulting in synergistic or competing impacts on its subcellular localization, stability, protein–protein interactions, DNA binding affinity, and transcriptional programs. Depending on the chemical nature and subcellular context, the oxidative-stress-mediated activation of FOXO3a can induce multiple transcriptional programs that play crucial roles in oxidative injury to the liver by chemicals. Here, we mainly review the role of FOXO3a in coordinating programs of genes that are essential for cellular homeostasis, with an emphasis on exploring the regulatory mechanisms and potential application of FOXO3a as a therapeutic target to prevent and treat liver oxidative injury.

## 1. Introduction

**Figure 1:**
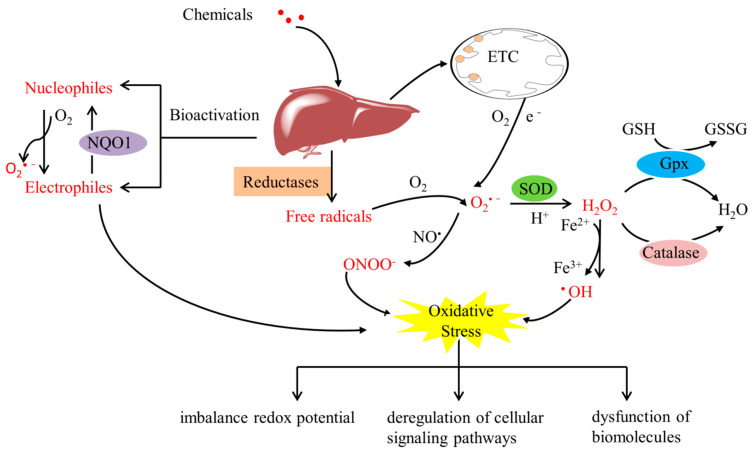
Reactive intermediate species metabolism induces oxidative stress in the liver. Liver oxidative injury is initiated by free radicals produced by metabolic conversion of chemicals into reactive intermediate species (red color), such as electrophilic compounds and ROS. Superoxide (O_2_^•−^) is generated as a by-product during oxidative phosphorylation within mitochondria. Superoxide can be converted to H_2_O_2_ by SOD enzymes. H_2_O_2_ is then scavenged by antioxidant enzymes such as GPx and catalase. ETC, electron-transport chain complexes; GPx, glutathione peroxidase; NQO1, NAD(P)H quinone oxidoreductase 1; SOD, superoxide dismutase.

Oxidative stress can be defined as an imbalance between antioxidant capacity and reactive oxygen species (ROS) production, and it has been increasingly acknowledged as a vital event in the hepatic deleterious effects of numerous chemicals such as alcohol, drugs, pesticides, and environmental pollutants, which can induce liver disorders such as nonalcoholic fatty liver disease (NAFLD), alcoholic liver disease (ALD), acute liver failure, and other liver injury [1,2]. As a key organ for the metabolism and detoxification of xenobiotics, the liver is also a preferential target of chemically induced oxidative insults and is vulnerable to oxidative injury. Over the past decade, liver oxidative injury by chemicals continues to increase as the main cause of acute hepatitis, posing a significant threat to public health. During the process of metabolism, many electrophilic compounds, reactive intermediate species, and free radicals are generated, disrupting the redox homeostasis and attacking biomolecules in the liver, resulting in irreversible damage (Figure 1) [3,4,5]. Among these reactive species, ROS, such as superoxide anions (O_2_^•−^), hydrogen peroxide (H_2_O_2_), and hydroxyl radicals (OH), have been widely implicated as crucial factors in determining the pathophysiology and development of xenobiotic-induced liver oxidative injury [6,7], although their mechanisms are still not fully understood. Oxidative stress can be assessed by indirectly measuring the level of ROS, including total cellular ROS and specific forms of ROS, such as indicators of mitochondrial superoxide, using chemical or genetically encoded redox-sensitive probes. Furthermore, a decrease in GSH level or in the GSH/GSSG ration can be used as an indicator of oxidative stress. Lipid peroxidation (in terms of MDA levels) and protein carbonyl content are common markers of cell oxidative damage and oxidative modification of proteins, respectively. In addition, the changes in the expression or activity of antioxidant enzymes such as SOD, catalase, and glutathione peroxidases (GPx) can also be viewed as markers of cellular response to oxidative stress. The activation of multiple signal pathways involved in oxidative injury coordinates the cellular response and ultimately determines the outcome.

Forkhead box O (FOXO) transcription factors are members of the forkhead box (FOX) family that have a highly conserved forkhead DNA-binding domain (DBD) [8]. FOXO transcription factors have been discovered in several species, including the worm (daf-16), the fly (dFoxO), zebrafish, rodents, and humans [9]. To date, four key elements of the mammalian FOXO subfamily have been found, including FOXO1, FOXO3a, FOXO4, and FOXO6. These proteins have a high level of profile similarity and vary mainly in their tissue-specific expression [10]. FOXO3a has been investigated extensively as a special and crucial regulator of cellular homeostasis, lifespan, and stress response. FOXO3a is regarded as a crucial regulator of many essential cellular processes, including cell proliferation, apoptosis, autophagy, and ROS detoxification [11,12,13,14,15]. FOXO3a’s subcellular distribution, protein–protein interactions, stability, and transcriptional selectivity may be influenced by ROS through post-translational modifications (PTMs) and epigenetic processes, as shown by accumulating data [16,17,18]. In this review, the functions of FOXO3a were summarized with emphasis on the regulation of FOXO3a, including PTMs and epigenetic mechanisms upon oxidative stress, as well as the underlying molecular mechanisms regulating its activity and functions in chemical-induced liver oxidative injury. Targeted interventions of FOXO3a signaling may provide promising therapeutic approaches against liver oxidative injury.

## 2. Architecture of FOXO3a Domains

**Figure 2:**
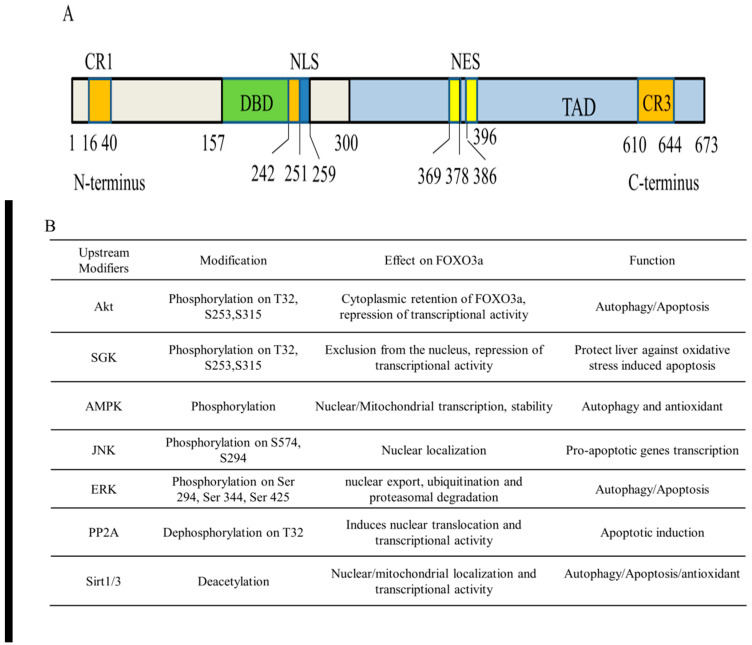
FOXO3a structure and its post-translational modifications to regulate liver oxidative injury. (**A**) Human FOXO3a domains. CR1–CR3, conserved regions 1–3; DBD: DNA-binding domain; NLS: nuclear localization signal domain; NES: nuclear export sequence; TAD, transactivation domain. (**B**) Major PTMs residues of FOXO3a regulated by various xenobiotics. In response to oxidative stress, FOXO3a undergoes PTMs in the NLS and NES domains, which affects its subcellular localization, stability, protein–protein interactions, and the transcriptional activity and specificity.

Human FOXO3a is ubiquitously expressed in the body, but varies in different types of cells, predominantly expressed in the liver [19]. The transactivation activity of FOXO3a is fine-tuned by four main domains necessary for the key locations of the PTMs, containing a nuclear localization signal (NLS), a nuclear export sequence (NES), an amino-terminal forkhead DNA-binding domain (DBD), and a C-terminal transactivation domain (TAD) (Figure 2). The N-terminal forkhead domain/DBD contains three helices (H1–H3), three β-strands (S1, S2, and S3), and two huge wing-like loops (W1, W2). The H3 helix region is the key component for DNA recognition and is directly engaged in binding the particular consensus DNA profile 5′-TTGTTTAC-3′ identified as the forkhead response element (FRE), according to crystal structure research [9,20,21]. Additionally, FOXO3a incorporates three conserved regions labeled CR1–CR3. The CR3 domain consists of 610–650 amino acids that are important for the enrollment of coactivators by identifying unique residues such as methylated arginine, likely causing FOXO3a to function as either a transcriptional activator or repressor upon DNA binding [22]. The NLS and NES domains regulate the subcellular localization of FOXO3a by binding to particular nuclear import and export receptor proteins, respectively. Consequently, the total FOXO3a conformation includes a hard head and a flexible tail [23].

Recent research suggests that FOXO3a works as a mitochondrial protein in preserving mitochondrial homeostasis in response to oxidative stress, which is rather intriguing. Mitochondrial FOXO3a (mtFOXO3a) is a cleaved FOXO3a isoform that loses residues 1–148 of the N-terminal domain but retains an entire DBD (amino acids 149–242). Therefore, mtFOXO3a can attach to mtDNA and stimulate the expression of mitochondrial genes. It is proposed that the FOXO3a N-terminus (aa 1–148) is crucially engaged in mitochondrial recruitment, with residues 98–108 being required for its cleavage and entry into the mitochondrial matrix [24].

## 3. Regulation of FOXO3a upon Oxidative Stress

FOXO3a is increasingly recognized as a central regulator of cellular homeostasis and oxidative stress response. FOXO3a activity is strictly and largely modulated by a variety of reversible PTMs in the NLS and NES domains that drive its translocation across subcellular compartments, therefore defining its inactivation in the cytoplasm or activation in the nucleus and mitochondria [25,26,27]. The most prevalent reversible FOXO3a PTMs include phosphorylation, acetylation, methylation, PARylation, and ubiquitination (Figure 2). The PTMs of FOXO3a can be recognized by their binding partners to regulate specific programs of gene expression in response to external stimuli, triggering synergistic or competitive effects at different levels, including stability, subcellular localization, DNA binding affinity, protein–protein interactions, and transcriptional activity [28,29,30,31]. Furthermore, emerging studies support the idea that FOXO3a employs epigenetic mechanisms such as histone modifications and microRNAs to control its own expression and activity [32].

Notably, oxidative modifications of FOXO3a seem important for models involving oxidative injury. In particular, reactive cysteine thiol groups of redox-sensitive transcription factor are revealed to be able to undergo rapid reactions with H_2_O_2_, thus forming sulfenic acid (-SOH) and other thiol oxidation products, such as the disulphide formed with nearby cysteines (-S-S-) [33]. Increasing evidence suggests that oxidative modifications of proteins may cause several changes, such as alterations in the proteins’ structure and function, changes in localization and physical interactions, and intervention in post-translational modifications such as phosphorylation [34]. However, currently, there are few reports regarding the oxidative modifications of FOXO3a in conditions of liver injury. In this review, we mainly review the role of FOXO3a as a transcription factor in the regulation of liver oxidative injury with a focus on discussing the regulatory mechanisms of FOXO3a, including PTMs and epigenetic mechanisms upon oxidative stress.

### 3.1. Post-Translational Modifications

The phosphorylation of FOXO3a has been implicated as an important fast response activated by ROS [35,36,37,38]. The most significant sensors of stress signals in the control of FOXO3a-dependent cellular homeostasis are PI3K and the 5′-AMP-activated protein kinase (AMPK) signaling mechanism. Additionally, the MAPK–FOXO3a axis has been found to be the primary homeostatic signaling pathway that controls the physiological reaction to oxidative stress.

Using H_2_O_2_ as a model oxidant, with the specific induction of cellular ROS, it has been shown that H_2_O_2_ triggers the stimulation of the PI3K mechanism, causing the phosphorylation of the downstream effector protein kinase B (PKB/AKT) at Ser473, which phosphorylates FOXO3a directly at three conserved residues (Thr32, Ser253, and Ser315). The activation of FOXO3a by Akt promotes its translocation to the cytoplasm and its interaction with the 14-3-3 nuclear export protein, thereby leading to the exclusion of FOXO3a from the nucleus, to cytoplasmic accumulation, and to proteasomal destruction. Therefore, Akt-mediated phosphorylation is a key suppressive PTM in the control of FOXO3a activity [39,40,41,42]. The serum and glucocorticoid-regulated kinase (SGK) is an additional downstream effector of PI3K activity. SGK is recognized to directly phosphorylate FOXO3a at sites that overlap with those identified by AKT, and then to stimulate the translocation to the cytoplasm and decrease FOXO3a activity [43,44].

In addition to Akt/SGK, AMPK, an important cell energy sensor and metabolic master, has also been involved in modulating the transcriptional activity of FOXO3a [45,46,47,48]. AMPK is a heterotrimer complex, including a catalytic subunit (α) and two regulatory subunits (β and γ). It is phosphorylated on the Thr172 in response to increased cellular AMP/ATP ratios due to the depletion of ATP during oxidative stress, allowing it to act as a rapid regulator of cellular energy homeostasis and control the redox state. Furthermore, it has also been proposed that exposure to H_2_O_2_ directly activates AMPK through S-glutathionylation of reactive cysteines located at the α- (Cys299 and Cys304) and β-subunits [49,50,51,52,53]. Thr179, Ser399, Ser413, Ser555, Ser588, and Ser626 may be directly phosphorylated by AMPK, resulting in the stimulation of FOXO3a transcriptional activity without influencing FOXO3a’s subcellular location [54]. The activation of AMPK also increases FOXO3a protein’s expression and stability under stress conditions and promotes nuclear FOXO3a and autophagy gene transcription [55]. Importantly, the activation of AMPK also results in FOXO3a phosphorylation on Ser 30 and induces FOXO3a translocation into mitochondria. FOXO3a in mitochondria may bind to mtDNA and react with mitochondrial transcription factor A (TFAM), mitochondrial RNA polymerase (mtRNApol), and SIRT3, inducing the production of various mitochondrial genes necessary for OXPHOS [24]. ROS have been identified as powerful inducers of the MAPK–FOXO3a axis, which regulates various cellular homeostatic processes [56,57]. FOXO3a, for instance, may be phosphorylated by stimulating mammalian Ste20-like protein kinase 1 (MST1), which disturbs the connection between FOXO3a and 14-3-3, hence boosting FOXO3a-dependent nuclear translocation and cell death [58,59,60]. Furthermore, the ERK-dependent phosphorylation of FOXO3a at Ser 294, Ser 344, and Ser 425 results in nuclear export, mouse double minute 2 homolog (MDM2)-mediated ubiquitination, and proteasomal degradation of FOXO3a, resulting in the inhibition of genes associated with cell death, such as Bcl2-like protein 1, Bim [61,62,63,64]. Similarly, the JNK cascade induces FOXO3a phosphorylation at Ser294 and Ser574 and promotes nuclear translocation, thus specifically potentiating the transcriptional activity of FOXO3a in apoptosis [65,66,67].

The dephosphorylation of FOXO3a mediated by protein phosphatase 2A (PP2A) at T32/S253 residues has a prominent function in directly regulating the nuclear translocation and transcriptional stimulation of FOXO3a, through inhibiting the dynamic interaction of the 14-3-3 protein with FOXO3a by Akt [68,69]. These studies clearly suggest that FOXO3a is regulated by an intricate network of phosphorylation and dephosphorylation to orchestrate the transcriptional control of a wide range of biological processes.

Furthermore, oxidative stress induces the acetylation/deacetylation of FOXO3a and influences its location and function. The histone acetyltransferase CREB binding protein (CBP) and its paralog p300 (CBP/p300) may acetylate FOXO3a, lowering its DNA-binding capacity and transcriptional activity. On the other hand, a class of stress-responsive histone deacetylases, such as SIRT1/3, is recognized to deacetylate and regulate the transcriptional action of FOXO3a at residues K242, K259, K290, and K569 [70,71,72,73,74,75].

Notably, the degradation of FOXO3a is also a significant way to adjust its role. The single molecule RING-finger E3 ligase MDM2 has been identified to trigger FOXO3a ubiquitination and degradation [64,76]. The inhibitory activity of the kinases, including IkappaB kinase (IKKβ), Akt, SGK, and ERK, triggers nuclear export and cytosolic sequestration, thus inhibiting its transcriptional activity and leading to its cytoplasmic proteasomal degradation. Therefore, the work demonstrates that FOXO3a localization in the cytoplasm not only disables FOXO3a activity, but is an important stage resulting in the degradation of FOXO3a.

Emerging evidence indicates that other PTMs, particularly methylation and the adding of poly (ADP-ribose) (PAR) chains (PARylation), influence FOXO3a activity. In mice, the stimulation of PRMT6 (protein arginine methyltransferase 6) has been found to promote the methylation of FOXO3a at Arg188 and Arg249, resulting in enhanced autophagy activity [77,78]. On the other hand, the PTM regulated by poly (ADP-ribose) polymerase-1 (PARP1) may be triggered by ROS and is implicated in the control of FOXO3a activity. PARP1 induces profound impacts on autophagy by boosting the nuclear accumulation and transactivation action of FOXO3a through suppressing the inhibitory phosphorylation that excludes FOXO3a from the nucleus [79]. Although reversible FOXO3a PTMs have been extensively studied in response to external stimuli, the coordination of regulators and the specificity are complex and warrant further investigation.

### 3.2. Epigenetic Regulation

**Figure 3:**
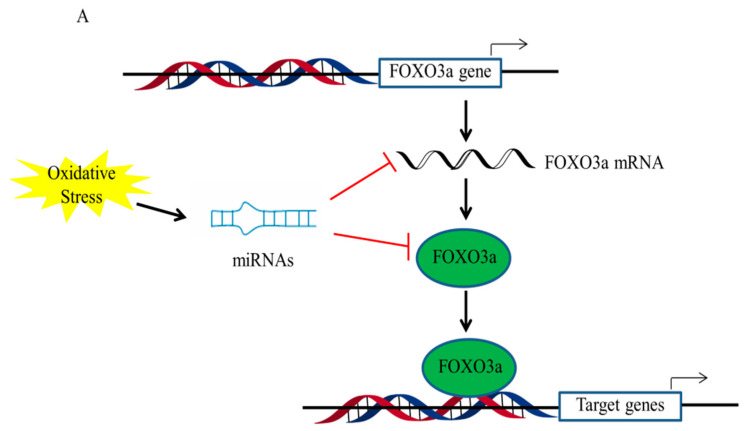

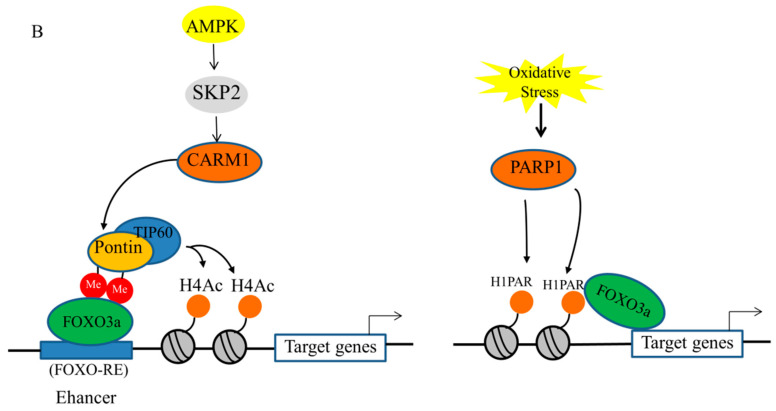
Epigenetic regulation of FOXO3a via microRNAs and histone modifications. (**A**). Diverse miRNAs are identified which regulate FOXO3a directly or indirectly upon oxidative stress. (**B**). Involvement of histone modifications in the control of FOXO3a transactivation in response to environmental factors. FOXO3a is responsible for autophagy gene expression by influencing chromatin structure through decrease in SKP2 to up-regulate CARM1, the CARM1–Pontin–FOXO3a signaling axis works for enhancer activation to establish target gene regulation by increasing H4 acetylation. Additionally, modification of histone H1 through PARylation by stimulation of PARP1 dissociates histone H1 from DNA, exposing the autophagy gene promoter regions and enhancing FOXO3a binding to the target gene promoters through epigenetic reprogramming of FOXO3a transactivation. CARM1: coactivator-associated arginine methyltransferase 1.

Recent evidence suggests that the epigenetic regulation of FOXO3a mainly includes microRNAs and histone modifications under oxidative stress conditions (Figure 3) [80,81]. MicroRNAs (miRNAs) have revealed new information on the function regulation of FOXO3a. Many miRNAs have been identified which directly or indirectly regulate FOXO3a. For instance, miR-155, miR-96, miR-223, and miR-21 are known to directly regulate FOXO3a, while miR-205 and miR-34a-5p regulate FOXO3a via the respective upstream targets, PTEN and Sirt3 [82,83,84]. Furthermore, it has been revealed that miR-378 in FOXO3a-regulated autophagy is associated with the kinase Akt, which facilitates the removal of malfunctioning or injured mitochondria by targeting the expression of the Akt stimulator, PDK1 (phosphoinositide-dependent protein kinase 1) [85]. A miR-Akt-FOXO3a axis has also been revealed in the liver, where miR-205-5p stimulates Akt and suppresses FOXO3a in primary hepatocytes, ultimately decreasing glucose synthesis [86].

Histone changes have been shown to be associated with the regulation of FOXO3a transactivation during interactions with environmental factors. A recent study stated that FOXO3a is responsible for regulating autophagy gene expression by suppressing SKP2 to up-regulate CARM1 [87]. FOXO3a was recruited to the FOXO response element (RE) containing genes upon starvation, and then methylated Pontin and Tip60 were co-recruited via the arginine methyl-binding residues along with increased H4 acetylation; that is, the CARM1–Pontin–FOXO3a signaling axis acts as a stimulation enhancer to establish target gene regulation by increasing H4 acetylation. It is also probable that these arginine methyl-binding residues are accessible to other methylated FOXO3a regulators, enabling appropriate and fine-tuned responses to various environmental stressors. Intriguingly, since FOXO3a plays crucial roles in stress responses, the discovery of new links between FOXO3a and methylated partners will provide the path for cells to adapt to varied pressures in novel ways.

The activity of PARP1 also serves as a DNA damage sensor by regulating the negative charge of histones and modulating histone–DNA interactions for chromatin remodeling, DNA repair, and transcription control [88]. Previous research demonstrated that the alteration of histone H1 by PARylation resulted in the separation of histone H1 from DNA, which exposed the promoter regions of autophagy genes and promoted FOXO3a binding to the promoters of target genes by the epigenetic reprogramming of FOXO3a transactivation [79]. These data indicate that epigenetic processes play a crucial function in controlling FOXO3a expression and subsequent transactivation of downstream targets; however, the epigenetic regulation of FOXO3a liver damage remains largely unknown.

## 4. Role of FOXO3a in the Liver

**Figure 4:**
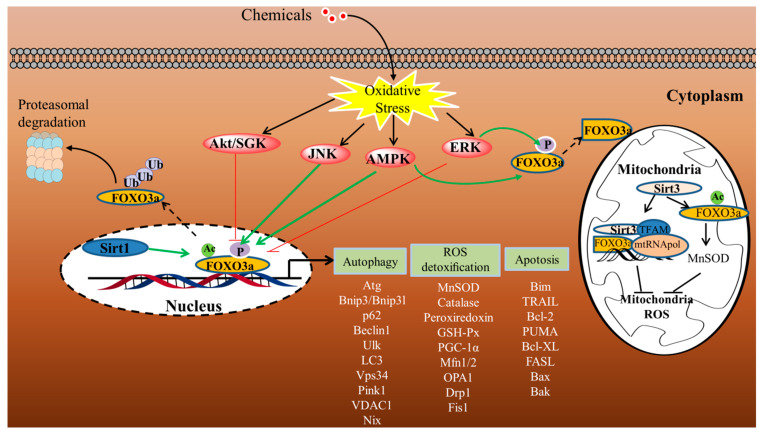
Proposed schematic representation of FOXO3a-mediated stress response in the liver. FOXO3a orchestrates multiple transcriptional programs to regulate apoptosis, ROS detoxification, and autophagy (arrows indicate active functions, and bar-headed lines represent inhibitory effects).

Accumulating evidence suggests that FOXO3a orchestrates the expression of genes related to cellular quality control and maintains cellular homeostasis under conditions of oxidative stress. Based on stress stimuli and the subcellular context, FOXO3a can cause particular groups of nuclear and mitochondrial gene expression in the liver, including autophagy effectors, pro-apoptotic genes and antioxidant genes. Here, we discuss the key roles of the FOXO3a–autophagy axis, FOXO3a-dependent apoptosis, and FOXO3a-regulated antioxidants in regulating liver oxidative injury (Figure 4).

### 4.1. FOXO3a-Autophagy Axis

Autophagy is a progressively preserved lysosomal degradation procedure that removes long-lived toxic aggregates of cellular proteins, lipids, injured organelles, and intracellular pathogens. Autophagy serves as a critical adaptive event in response to changed cellular signaling or stresses and has a crucial function in cellular renovation and preservation of cellular homeostasis [89,90,91,92,93,94]. FOXO3a has been implicated in autophagy in a variety of cells to protect them from various stresses [79,80,95,96]. Notably, several studies have revealed that FOXO3a induces the expression of numerous autophagy genes, such as genes related to autophagy initiation (Atg101, Ulk1/2), vesicle nucleation (Atg14, Vps34), elongation (LC3b, Atg5), and mitophagy (Bnip3, Beclin1, Pink1), through binding to the promoter regions and transactivating the expression of autophagy genes in response to oxidative stress.

Ethanol treatment has the potential to produce excessive ROS in hepatocytes, particularly superoxide (O_2_^•−^), and to elicit significant liver oxidative injury, such as steatosis, inflammation, fibrosis, and cirrhosis, through mitochondrial damage and endoplasmic reticulum stress. Interestingly, it has been revealed that acute ethanol treatment modulates autophagy as a compensatory pathway to mitigate ethanol-induced liver injury [97]. Acute ethanol treatment significantly increased the expression of many key autophagy-related genes, including Ulk1, Atg5, Beclin1, Bnip3, Bnip3L, Atg7, LC3b, Atg14, and Vps34, which were induced by nuclear translocation of FOXO3a in primary cultured mouse hepatocytes and in the liver. Multiple PTMs, such as Akt- and Sirt1-mediated reduced phosphorylation and enhanced deacetylation of FOXO3a, were discovered to defend against alcohol-induced liver damage through nuclear translocation and transcriptional regulation of those autophagy-related genes. FOXO3a^−/−^ mice treated acutely with ethanol demonstrated lower expression of autophagy-related genes, but elevated liver damage. These findings indicate that FOXO3a is a crucial factor in regulating in vitro and in vivo ethanol-induced autophagy and cell survival [98]. Furthermore, another study indicated that the Farnesoid X Receptor (FXR) mediated the stimulation of FOXO3a in ethanol-induced autophagy and hepatotoxicity. Acute alcohol treatment in FXR KO mice was found to stimulate Akt, enhance FOXO3a phosphorylation, and reduce FOXO3a nuclear retention, in addition to the transcription of autophagy genes Atg5, Becn-1, and MAP1LC3B, thereby inducing hepatic mitochondrial spheroid formation, which may be utilized as a compensatory substitute mechanism to eliminate damaged mitochondria induced by ethanol [99]. According to these findings, the lack of FXR disrupted FOXO3a-mediated autophagy, which, in turn, increased alcohol-induced liver damage. Furthermore, the AMPK–FOXO3a axis has been revealed to regulate autophagy-related genes, including Beclin-1 and LC3B, in both primary rat hepatocytes and human liver cells [100].

Mitophagy, the selective destruction of damaged mitochondria by autophagy, is necessary to maintain healthy mitochondria. Mitophagy dysfunction in hepatic cells has been identified in several liver disorders [101]. Defective mitophagy leads to increased ROS production, ATP depletion, apoptosis-related protein production, and dysregulated stress signaling transduction [102]. By inhibiting mitophagy, 2,2′,4,4′-tetrabromodiphenyl ether (BDE-47) has been shown to induce mitochondrial dysfunction, redox state imbalance, and accompanying liver oxidative damage. Evident liver injuries were observed in BDE-47-treated mouse livers, and the ROS production and MDA content were markedly increased, while the expression and activity of mitochondrial antioxidative enzyme MnSOD were notably decreased in the livers. These results indicate that BDE-47 induces mitochondrial dysfunction and related liver oxidative injury in mice. Additionally, in the livers of mice supplemented with BDE-47, Parkin, an E3 ubiquitin ligase, was significantly down-regulated. Furthermore, BDE-47 dramatically decreased both the LC3II/LC3I ratio and the expression of mitochondrial LC3II protein. Furthermore, this research demonstrated that BDE-47 significantly inhibited the expression and activity of Sirt3, resulting in a substantial rise in the protein expression of Ac-FOXO3a and a reduction in the protein expression of PINK1 in vivo. Notably, miR-34a-5p significantly inhibited Sirt3/FOXO3a/PINK1-mediated mitophagy in BDE-47-treated mouse liver to enhance mitochondrial dysfunction and hepatotoxicity [82]. These results demonstrate that FOXO3a is essential for the control of mitophagy to protect the liver from xenobiotic-induced oxidative stress.

A study by Zhou Y. et al. discovered FOXO3a to be a direct downstream target of miR-223, mediating the decrease in the LC3-II/LC3-I ratio and the elevation of p62 expression, leading to the suppression of doxorubicin-induced autophagy in hepatic cells [84]. Interestingly, FOXO3a also has been implicated in the promotion of a specific form of autophagy known as lipophagy in the liver. In vitro and in vivo analyses established that FOXO3a could positively regulate Atg14 gene expression via a reaction with the cis-elements of proximal insulin response elements (IRE) [103]. Notably, the expression of many autophagy-related genes, such as LC3B, Gabarapl1, Bnip3, and Bnip3l, can contribute to FOXO3a in the circadian induction of autophagy. In a recent work, the authors found that insulin controls the molecular clock in a PI3K- and FOXO3a-dependent method, suggesting a critical function for the insulin-FOXO3a-clock signaling mechanism in the regulation of circadian rhythms [104]. FOXO3a is also implicated in connecting the circadian clock to metabolism in the mouse liver [105]. All these studies suggest that the FOXO3a-autophagy axis is pivotal in regulating liver oxidative injury. Although it is well recognized that FOXO3a can directly cause the expression of autophagy genes throughout its transactivation processes in xenobiotic-induced liver injury, the regulation of its own gene expression and the underlying mechanisms of specificity based on the stress trigger and physiological context are widely undefined.

### 4.2. FOXO3a-Regulated Apoptosis

Apoptosis is a spontaneous and orderly programmed cell death modulated by related genes, resulting in the self-elimination of excessively damaged or nonfunctional cells [106]. Oxidative stress can trigger excessive apoptosis by modifying critical cellular components via the mitochondrial pathways [107]. Multiple signaling mechanisms are involved in oxidative-stress-induced hepatocyte death, including the ERK1/2, SGK, JNK, and FOXO3a signaling mechanisms. The suppression of autophagy enhances the accumulation of FOXO3a and the transactivation of many proapoptotic genes by FOXO3a in response to oxidative stress. There is evidence that FOXO3a is a crucial transcriptional regulator of Bim and PUMA expression. Due to its ability to bind to and neutralize all prosurvival Bcl-2 members, it is regarded as the most effective of the proapoptotic BH3-only proteins. Even so, the particular cell signal mechanism differs based on the intensity and period of oxidative stress as well as the cell type, and the underlying mechanisms of the balance between proapoptotic and pro-survival activities of FOXO3a remain obscure. A better understanding on how FOXO3a-dependent apoptosis is differentially controlled in the liver may provide insight into the etiology of xenobiotic-induced liver oxidative damage.

Wnt/β-catenin and FOXO3a have been revealed to have a crucial function in protecting the liver versus 3,5-diethoxycarbonyl-1,4-dihydrocollidine (DDC) and paraquat [108]. The stimulation of Wnt/β-catenin signaling inhibits FOXO3a-induced cell death by up-regulating the β-catenin target gene serum/glucocorticoid regulated kinase 1 (SGK1). Conversely, SGK1 was considerably reduced, which prevented it from inactivating FOXO3a, leading to the nuclear retention of FOXO3a and elevated proapoptotic target gene expressions of p27 and Bim in β-catenin KD livers exposed to oxidative stress. In addition, the removal of FOXO3a boosted hepatocyte resistance to oxidative-stress-induced apoptosis, validating FOXO3a’s proapoptotic involvement in the stressed liver. These data imply that the phosphorylation of FOXO3a by SGK1 inhibits its apoptotic activity, hence increasing hepatocyte survival [108].

Ac-FOXO3a is more likely to cause cell death, while deacetylated FOXO3a exhibits activated transcriptional function and antioxidant potential [66]. Recent research suggests that the environmental contaminant hexavalent chromium (Cr(VI)) generated an elevation in acetylated FOXO3a by suppressing Sirt1 expression and activating the Bim/PUMA axis, culminating in oxidative-stress-mediated death in hepatocytes. Treatment with resveratrol, a Sirt1 activator, significantly reduced acetylation and reversed liver damage, indicating that resveratrol may have a therapeutic effect on Cr(VI)-induced liver injury. In contrast, suppression of Sirt1-mediated deacetylation of FOXO3a exacerbates oxidative stress and the development of Cr(VI)-induced hepatotoxicity. These investigations established the connection between acetylation of FOXO3a and apoptosis triggered by environmental contaminants, establishing the foundation for a more complete comprehension of chemical hepatotoxicity [109].

Interestingly, FOXO3a expression is highly associated with enhanced cell death in the liver of chronic ethanol-fed rats and negatively related with suppressed β-catenin signaling. Ethanol exposure reduced the phosphorylation of FOXO3a and increased nuclear localization of FOXO3a, as well as the exhibition of liver injury and apoptosis in rats. The proapoptotic protein Bim, a downstream target gene of FOXO3a, was up-regulated, together with antiapoptotic signals modulated by Bcl-2, Bcl-XL, and pro-caspase 3 which were inhibited in the liver. Moreover, it demonstrated that SGK1 functional kinase activity, but not Sirt1, was needed for FOXO3a-induced apoptosis. Chronic ethanol consumption inhibited β-catenin signaling and resulted in SGK1 expression reduction, which in turn supported FOXO3a increase to cause hepatocyte death, suggesting that FOXO3a has a key function in promoting the death of hepatocytes [110].

Although it is known that the dephosphorylation of FOXO3a caused by a decrease in SGK1 in response to alcohol exposure promotes the expression of proapoptotic genes over antioxidant genes, the precise locations necessary for this interaction remain unknown. Recent evidence indicates that ethanol stimulates the JNK-dependent phosphorylation of FOXO3a at serine-574, and that p-574-FOXO3a preferentially attaches to promoters of proapoptotic genes but not to antioxidant targets in hepatocytes, showing that p-574-FOXO3a is exclusively pro-apoptotic. The Bcl-2 promoter was bound by both unphosphorylated and p-574-FOXO3a, but the unphosphorylated form was a transcriptional activator and the p-574-FOXO3a form was a transcriptional repressor. In addition, targeted alterations at S-574 reveal that the charge at this position is a crucial determinant of FOXO3a’s proapoptotic action. This research indicates that S-574 phosphorylation creates a particularly apoptotic form of FOXO3a after ethanol treatment in hepatocytes [67]. Interestingly, a recent study has demonstrated that acute ethanol gavage induced FOXO3a-dependent Kupffer cell apoptosis in mice and subsequently protected against ethanol-induced liver injury via attenuating the liver pro-inflammatory phenotype mediated by promoting infiltrating macrophage differentiation [13]. Although the finding explored the link between FOXO3a and inflammation in xenobiotic-induced liver injury, a better understanding of the potential pathways remains to be determined.

In addition, the transcriptional regulation of Bim by FOXO3a has also been implicated in lipoapoptosis in some hepatocytes treated with saturated free fatty acids (FFA). FFAs induced FOXO3a dephosphorylation/activation by protein phosphatase 2A (PP2A), but not a reduction in the phosphorylated form of Akt and SGK in Huh-7 cells, HepG2 cells, and murine hepatocytes, highlighting the PP2A–FOXO3a–Bim pathway as a critical toxicity pathway in the regulation of apoptosis [111]. A similar study showed that the decrease in the phosphorylation of FOXO3a at Thr32, modulated by stimulation of the phosphatase PP2A, which is needed for 14-3-3 binding, suppressed FOXO3a turnover, caused a nuclear gathering of p53, inhibited cytoplasmic p53, and suppressed mitochondrial-mediated cell death. These data indicate that interactions between p53, FOXO3a, and 14-3-3 lead to reduced benzo[a]pyrene (BaP)-caused death in cells co-exposed to TCDD, PCB 153, or estradiol, and targeting FOXO3a might thus damage a cell’s capability to carefully handle xenobiotics via the weakening of cell death [112].

Although autophagy and apoptosis have been extensively investigated in xenobiotic-induced liver injury, the coordination and interplay between FOXO3a and the processes of oxidative damage are complicated and are not fully understood. In general, both autophagy and apoptosis as partners affect each other, and autophagy tends to be antiapoptotic by elevating the cutoff point of stress needed to cause apoptosis [113]; however, the mechanisms that determine the basal grades of autophagy and the cutoff point for death are still in their infancy. Taken together, these studies establish a dual autophagy–apoptosis regulatory role of FOXO3a to maintain cellular homeostasis to further regulate liver oxidative injury, but the interaction between the intrahepatic signaling response and FOXO3a transcriptional programs of different xenobiotics, as well as the specific mechanisms, need further investigation.

### 4.3. FOXO3a-Mediated Regulation of the Antioxidant System

Excessive generation of ROS has been widely implicated in the etiology and progression of liver oxidative injury by various chemicals [114,115,116,117]. An increasing number of studies show that the FOXO3a signaling pathway protects hepatocytes from oxidative damage by stimulating the transcription of genes coding for multiple antioxidants and ROS detoxification. Many antioxidant enzymes, such as GSH-Px, MnSOD, Peroxiredoxin (Prx), Catalase, and mitochondrial oxidative phosphorylation (OXPHOS), have been found to be up-regulated at the transcriptional level upon activation of the FOXO3a pathway through the direct or indirect binding of FOXO3a to the promoters of these target genes in the nuclei or mitochondria. Studies show that MnSOD catalyzes dismutation of superoxide to generate oxygen and hydrogen peroxide (H_2_O_2_). H_2_O_2_ is further dismutated to water and oxygen in a reaction catalyzed by a peroxisomal heme peroxidase, called catalase. Alternatively, peroxiredoxins are also the major cellular enzymatic scavengers that control H_2_O_2_ level, which are known gene targets of FOXO3a. The up-regulation of these antioxidant enzymes can reduce cellular ROS production, consequently ameliorating oxidative injury, and raise cellular survival in the stressed liver [98,118,119,120,121]. In this respect, a better understanding is necessary for the function of FOXO3a in mediating nuclear–mitochondrial crosstalk because they can influence each other’s activities, such as oxidative stress response.

On the other hand, mitochondria homeostasis preserves the role and integrity of the mitochondria by coordinating its biogenesis and fusion–fission dynamics involving FOXO3a-dependent pathways. FOXO3a controls ROS metabolism by suppressing the expression of a set of nuclear-encoded mitochondrial genes by stimulating the expression of MAX dimerization proteins (MAD/MXD) and modifying the stability/function of the c-MYC protein. In addition, FOXO3a stimulation reduced the mtDNA copy number, the expression of mitochondrial proteins, and the levels of respiratory complexes, therefore significantly lowering ROS formation [122]. Peroxisome proliferator-activated receptor γ coactivator 1α (PGC-1α) is a well-characterized master regulator of mitochondrial biogenesis and a set of genes related to mitochondrial function and oxidative metabolism. Significantly, FOXO3a may protect cells from oxidative stress by interacting directly with PGC-1α. FOXO3a and PGC-1α are recruited to the identical promoter regions and trigger a set of antioxidative genes [123], as shown by co-immunoprecipitation and in vitro interaction experiments. In addition, FOXO3a is a direct transcriptional regulator of PGC-1α, indicating that an auto-regulatory loop controls the FOXO3a/PGC-1α regulation of mitochondrial oxidative stress protection [123].

Recent research has demonstrated that the Sirt3-mediated deacetylation of FOXO3a positively modulates related genes in order to coordinate mitochondrial fission and fusion. Mitochondrial fusion is induced by mitofusin 1 (Mfn1), Mfn2, and optic atrophy 1 (OPA1) to mediate the repair of damaged mitochondrial DNA, whereas mitochondrial fission is regulated by dynamin-related protein 1 (Drp1) and Fis1 to initiate the separation of damaged mitochondria from healthy mitochondria. Mitochondrial fission and fusion can be targeted for degradation, the consequences of which improve mitochondrial efficiency and cellular tolerance to oxidative damage involving FOXO3a in liver oxidative injury caused by chemicals such as senecionine [124,125,126,127].

Overall, FOXO3a functions as a pivotal transcription factor responsible for several transcriptional programs such as cell survival and death. Nonetheless, the precise mechanisms of FOXO3a that govern transcriptional program specificity need to be further investigated, including the basal level of protein expression, different cell types of the liver, special PTMs, epigenetic regulation, key cofactors, stress stimuli, and so on. Moreover, although increased ROS formation has been widely acknowledged as a crucial mechanism underlying the cytotoxicity and liver injury caused by various chemicals, other mechanisms beyond ROS may also be critically involved in FOXO3a-mediated effects for many chemicals.

## 5. FOXO3a as a Potential Therapeutic Target

A growing amount of evidence recognizes FOXO3a as a promising protective target in preventing or reversing xenobiotic-induced liver oxidative injury. To date, many natural products, such as saponins, flavonoids, anthraquinone, and polyphenols, have been shown to efficiently protect the liver from oxidative injury by targeting FOXO3a (Table 1). Resveratrol, a well-known Sirt1 agonist, exerts multiple pharmacological effects such as anti-inflammatory, antioxidant, cardioprotective, and anti-aging effects. It has been found to enhance the ethanol-prompted expression of autophagy-related genes Atg5, LC3b, Vps34 and autophagosome formation by increasing the Sirt1-mediated deacetylation of FOXO3a, resulting in protection against ethanol-induced liver damage [98]. Resveratrol deacetylates FOXO3a and modulates its gene transcription and specificity, which increases the expression of antioxidative genes in a target-specific manner in response to oxidative stress. However, as resveratrol is a non-specific compound, detailed studies on the potential toxicity are clearly needed. Although orally administered resveratrol, at doses of 200 mg/kg/day in rats and 600 mg/kg/day in dogs for 90 days, did not show obvious toxic effects, others reported systemic inhibition of P450 cytochromes in high doses. The hepatoprotective effects of zeaxanthin dipalmitate (ZD), a lipophilic antioxidant, have been evaluated in both in vitro and in vivo investigations. It is revealed that the direct targets of ZD on the cell membrane contain receptor P2X7 and adiponectin receptor1 (adipoR1). Signals from P2X7 and adipoR1 control the PI3k–Akt and AMPK–FOXO3a mechanisms to regain ethanol-inhibited mitophagy activities. Intriguingly, cotreatment with ZD restored the normal translocation of FOXO3a from the cytosol to the nucleus, but ZD therapy alone enhanced the basal nuclear translocation of FOXO3a [128]. A bioactive antioxidant polyphenol found in pomegranates, Punicalagin (PU), exhibited antioxidant, anti-inflammatory, hepatoprotective, and antigenotoxic activities and simultaneously indicated low toxicity. A recent study revealed that PU protects human hepatocytes and mouse liver from CCl_4_-induced oxidative injury through the up-regulation of antioxidative activities and activation of autophagy mediated by the Akt/FOXO3a signaling pathway [129]. Additionally, quercetin, a naturally occurring flavonoid, exerted prominent anti-oxidative and anti-inflammatory activities. It could scavenge free radicals and protect cells from hydrogen peroxide damage. Since this flavonoid has no obvious adverse effects, it is generally considered safe. Quercetin was found to protect the liver from ethanol-induced mitochondrial damage through activating mitophagy-mediated enhancement of mitophagosomes-lysosome fusion and the transcriptional activity of FOXO3a via the AMPK and ERK2 signaling pathways [130]. Melatonin, a pineal-gland-produced indolamine, is a strong mitochondria-targeted antioxidant and free radical scavenger, with uncommonly high safety profile. It has been demonstrated to protect against sodium fluoride (NaF)-induced hepatotoxicity by up-regulating Sirt3 expression levels and enhancing the activity and expression of SOD2 through Sirt3-regulated transcriptional activity of FOXO3a, thus inhibiting oxidative damage [121]. The translocation of FOXO3a into the nucleus also has a vital function in regulating pharmacological-agents-mediated protection effects and autophagy. For instance, globular adiponectin (gAcrp) has been found to induce the translocation of FOXO3a into the nucleus and to trigger the expression of genes associated with autophagy, such as LC3, beclin-1, and Atg5 in both primary rat hepatocytes and human hepatoma cell lines. Additionally, the AMPK–FOXO3a axis has been involved in the stimulation of autophagy by adiponectin and the consequent prevention of ethanol-induced apoptosis [100,131].

Taken together, these findings suggest that FOXO3a exerts promising therapeutic value in xenobiotic-induced liver oxidative injury, in spite of the difficult interference from the high-affinity binding of small molecules, due to the fact that the structure of FOXO3a transcription factor is a relatively flat surface. Further investigation of specific pharmacological discoveries depending on the effective amelioration of hepatic oxidative stress resistance, autophagy, inflammation, and apoptosis via the selectivity and specificity regulation of FOXO3a transcriptional activity will be a challenge and could result in further novel methods for the treatment of liver oxidative injury.

## 6. Conclusions and Implications

The FOXO3a transcription factor has been extensively investigated as a pivotal mediator in many key cellular processes. Importantly, FOXO3a orchestrates multiple transcriptional programs to maintain cellular homeostasis in the stressed liver by not only targeting pro-survival genes but also by targeting those involved in promoting apoptosis. In fact, different types of stressors can differentially modulate the FOXO3a-mediated stress response to achieve the adaptive regulation of cellular homeostasis by different combinations of upstream stress regulators that are physically able to control nuclear and mitochondrial FOXO3a activity through various PTMs and epigenetic mechanisms. The key points of the processes involve the following: (i) The function of FOXO3a is strongly controlled by multiple PTMs, such as phosphorylation, acetylation, ubiquitination, and PARylation, and further investigations into the epigenetic mechanisms that regulate FOXO3a are necessary to explain its function in response to oxidative stress in the liver. Specific PTMs govern the partition, stability, and degradation of FOXO3a, but less is understood about how FOXO3a PTMs define the specificity of its transcriptional program. Specific PTMs are sufficient to induce a transcriptionally selective form of FOXO3a, which reveals that the prospect of altering the regulatory pathway might be a significant treatment option for xenobiotic-induced liver damage. (ii) Numerous experiments reveal that FOXO3a orchestrates different transcriptional programs related to many cellular processes, such as cell death, ROS detoxification, and autophagy, either by promoting apoptotic signaling cascades such as Bim, Fas ligand, or TRAIL under elevated stress or by antagonizing oxidative stress by triggering autophagy and stress resistance genes under low stress, depending on the cellular context, the modification pattern, the oxidative stress conditions, and so on. However, the complex coordination of autophagy and apoptosis, and the underlying mechanisms for balance, are not yet completely understood. (iii) It is becoming increasingly evident that the roles of FOXO3a in mediating nuclear–mitochondrial crosstalk are critical for the restoration and preservation of cellular homeostasis as they can influence each other’s activities; however, this interaction remains poorly characterized, and further work on the topic will help to explain the level of complexity of stress-activated pathways. (iv) The interplay between FOXO3a and other transcription factors or regulatory mechanisms such as p53, inflammatory response, and mTOR signaling needs further attention in liver oxidative injury research. Collectively, the interplay between autophagy, apoptosis, the antioxidant defense system, and other regulatory pathways controlled by FOXO3a results in the dynamic homeostasis of cell survival or apoptosis processes in response to xenobiotic-induced liver injury.

Given the complexity of FOXO3a-mediated gene regulatory systems, more investigations are needed to elucidate FOXO3a’s role as an effective therapeutic target able to prevent or limit the development of liver oxidative injury. Further studies on the transcriptional activity and specificity, as well as the kinetics of induction, will expand our understanding of how FOXO3a functions to have an effect on homeostasis and be critical for the progress of novel treatment targeting the FOXO3a signaling axis in liver oxidative injury.

## Figures and Tables

**Table 1:** Selected compounds known to affect FOXO3a activity.

Compounds	Target	Structure	Key Events Regulated by FOXO3a
Melatonin	Sirt3	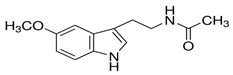	Increases the expression of SOD2
Punicalagin	Akt	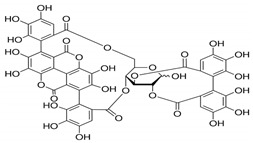	Increases antioxidative activities and autophagy
gAcrp	AMPK		Induces autophagic process and inhibits apoptosis
Resveratrol	Sirt1	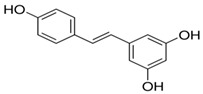	Enhances expression of autophagy-related genes and autophagosome formation
Zeaxanthin dipalmitate	P2X7 adipoR1	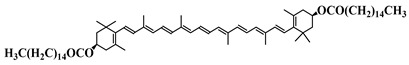	Modulates PI3k–Akt and AMPK–FOXO3a pathways to restore mitophagy function
Quercetin	AMPK ERK2	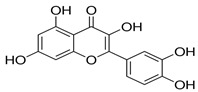	Activates mitophagy

## Data Availability

Not applicable.
